# Supplementary material for: Visible colorimetric dosimetry of UV and ionizing radiations by a dual-module photochromic nanocluster
Source: Nat Commun. 2021 May 14;12:2798. doi: 10.1038/s41467-021-23190-0 (PMC8121945; doi:10.1038/s41467-021-23190-0)
Supplement: Supplementary file 1 — Supplementary Information [file 41467_2021_23190_MOESM1_ESM.pdf]

Supplementary Information for

Visible Colorimetric Dosimetry of UV and Ionizing Radiations by a Dual-Module Photochromic Nanocluster

Lu and Xie et al.

Supplementary Figures

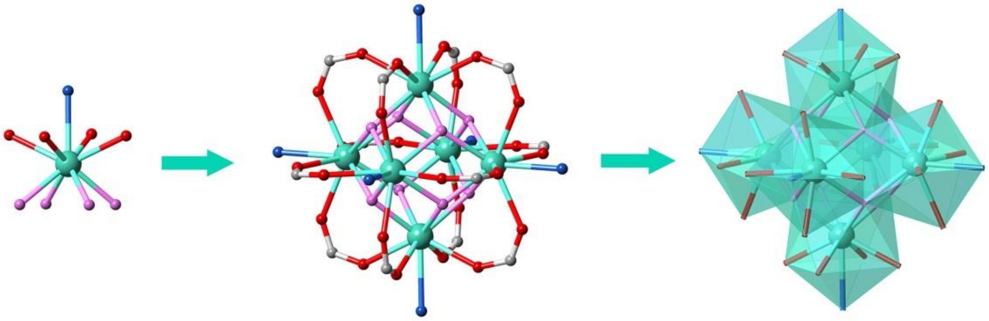

Supplementary Figure 1. Structure of the hexameric $[Th_6(OH)_4O_4(H_2O)_6]^{12+}$ core. The Th atoms are shown in green; carbon atoms are shown in gray; and the O atoms from μ_3-OH/-O, H_2O, and bridging carboxylate groups are shown in pink, blue, and red, respectively.

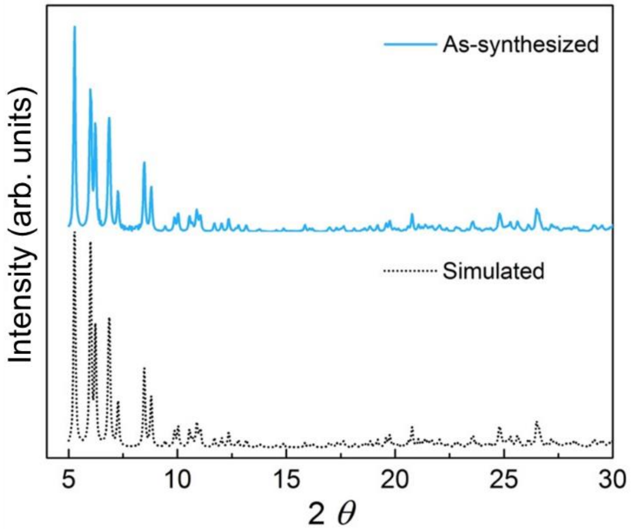

Supplementary Figure 2. PXRD patterns of simulated and as-synthesized Th-SINAP-100 samples.

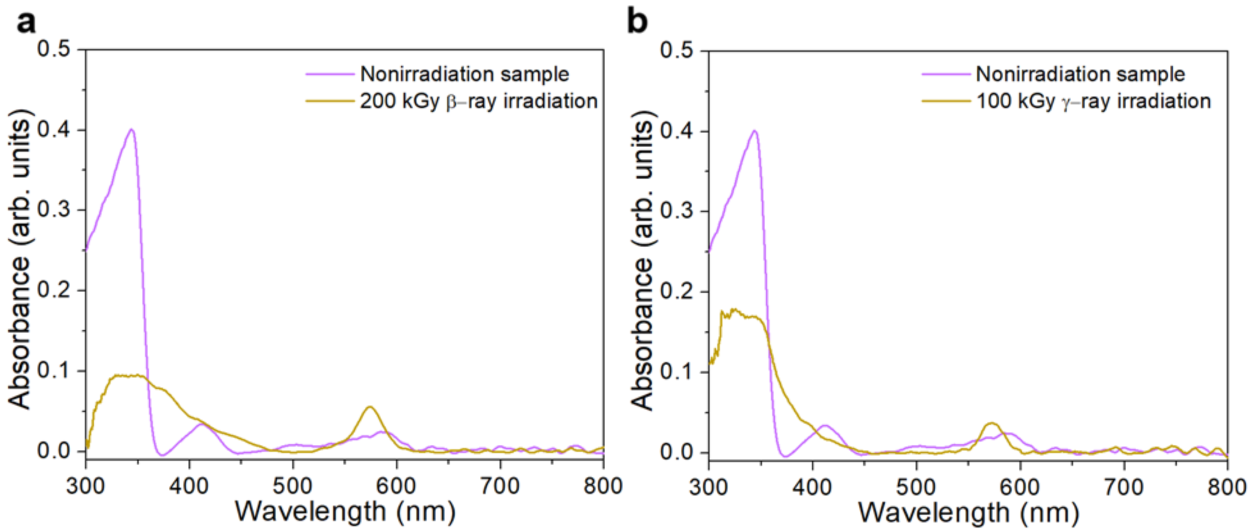

Supplementary Figure 3. Solid-state UV-Vis absorption spectra of Th-SINAP-100 crystal before and after (a) β and (b) γ-ray irradiations. The UV-Vis spectra were collected on a crystal of Th-SINAP-100 from 300 to 800 nm based on the average intensities of 50 scans with a scan time of 2 ms.

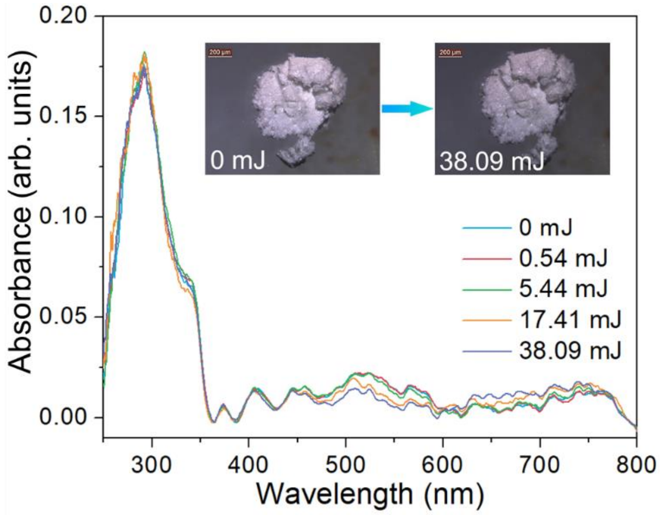

Supplementary Figure 4. Evolution of the solid-state UV-Vis absorption spectra and color of HTPC from 0 to 38.09 mJ upon UV irradiation (λ_{ex} = 365 nm, 2 mW). The UV-Vis spectra were collected on HTPC powder from 250 to 800 nm based on the average intensities of 50 scans with a scan time of 2 ms.

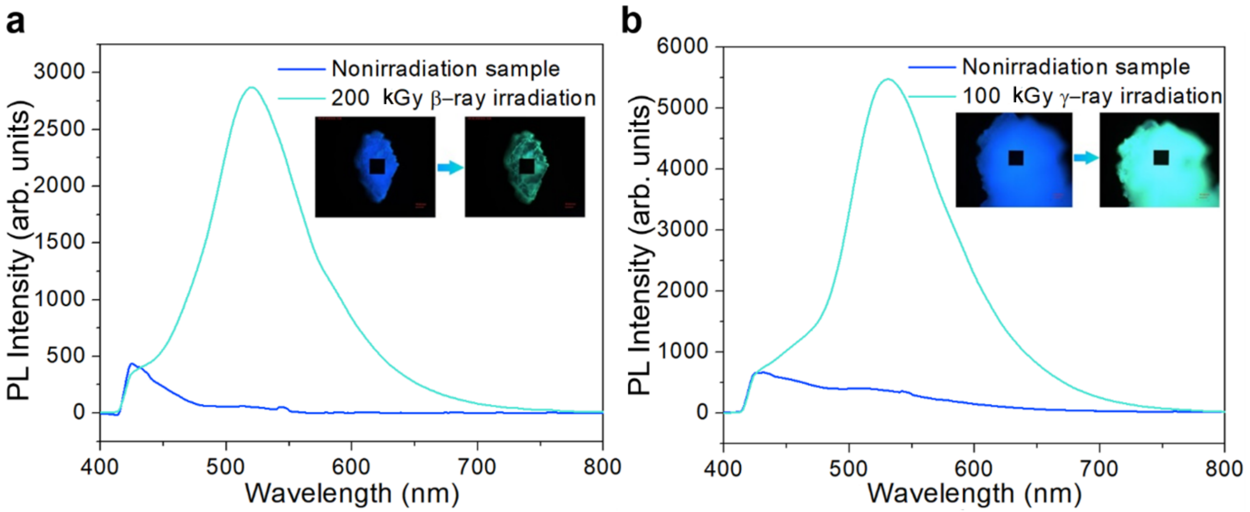

Supplementary Figure 5. Solid-state photoluminescence (PL) spectra (λ_{ex} = 365 nm, 2 mW) and colors of Th-SINAP-100 crystal before and after (a) 200 kGy doses of β-ray and (b) 100 kGy doses of γ-ray irradiation. The PL spectra were collected on a crystal of Th-SINAP-100 from 400 to 800 nm based on the average intensities of 5 scans with a scan time of 500 ms.

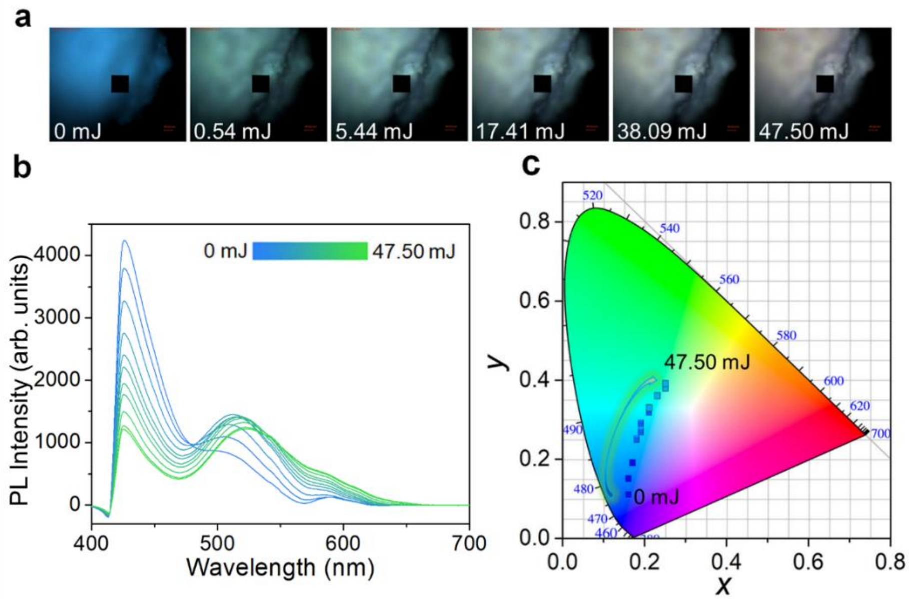

Supplementary Figure 6. (a) Snapshots of HTPC upon 365 nm UV irradiation. (b) Evolution of the solid-state PL spectra of HTPC upon 365 nm UV irradiation (λ_{ex} = 365 nm, 2 mW). The PL spectra were collected on HTPC powder from 400 to 700 nm based on the average intensities of 5 scans with a scan time of 500 ms. (c) CIE chromaticity coordinates of HTPC as a function of UV irradiation dose.

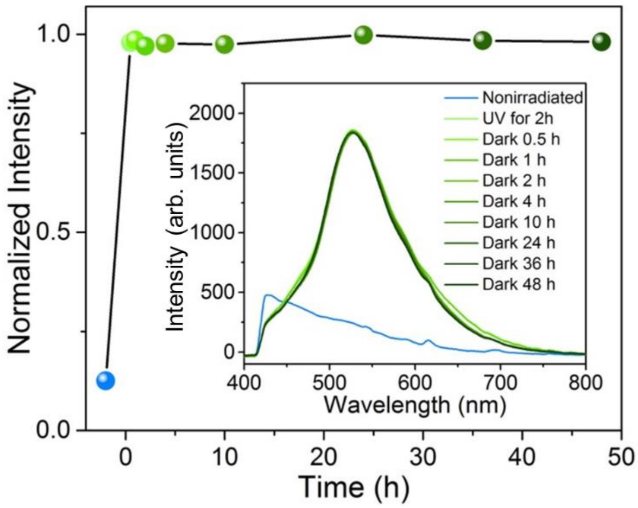

Supplementary Figure 7. The stability measurement of UV radiation induced radicals. Inset: the solid-state PL spectra (λ_{ex} = 365 nm, 2 mW) of Th-SINAP-100 crystal showing that PL modulation is not reversible under ambient conditions. The PL spectra were collected on a crystal of Th-SINAP-100 from 400 to 800 nm based on the average intensities of 5 scans with a scan time of 500 ms.

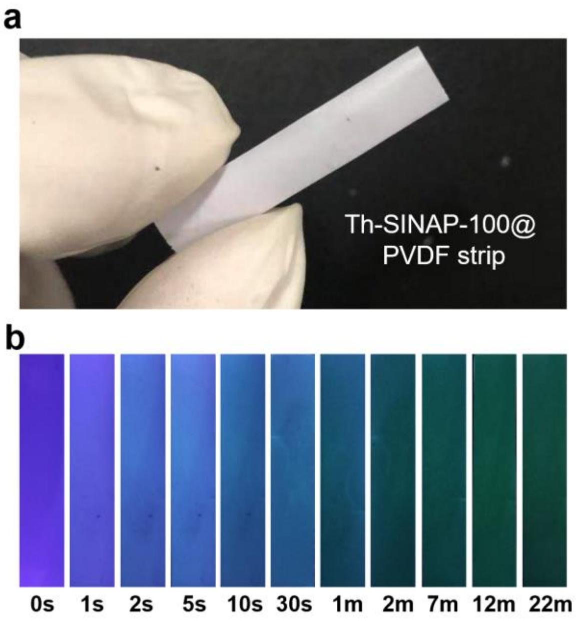

Supplementary Figure 8. (a) Photograph of a Th-SINAP-100@PVDF strip. (b) An illustration of Th-SINAP-100@PVDF strip functioning as a radiation dosimeter.

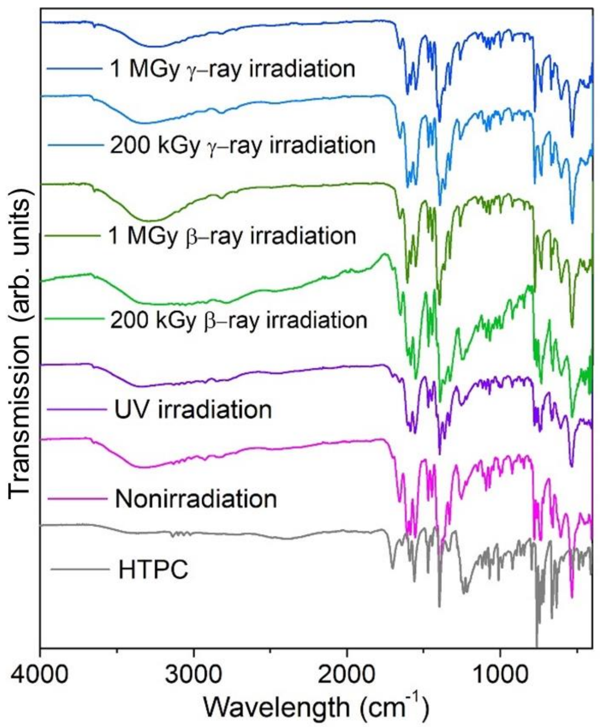

Supplementary Figure 9. FTIR spectra of Th-SINAP-100 before and after irradiation with UV, β-, and γ-ray.

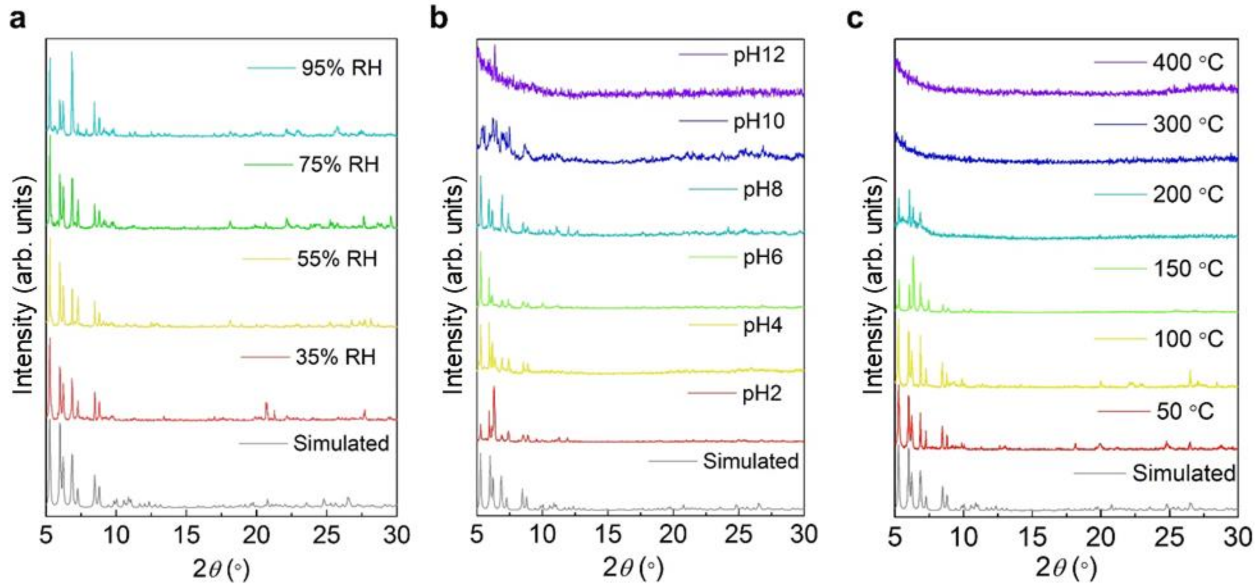

Supplementary Figure 10. PXRD spectra of Th-SINAP-100 under different (a) RH, (b) pH, and (c) temperature conditions.

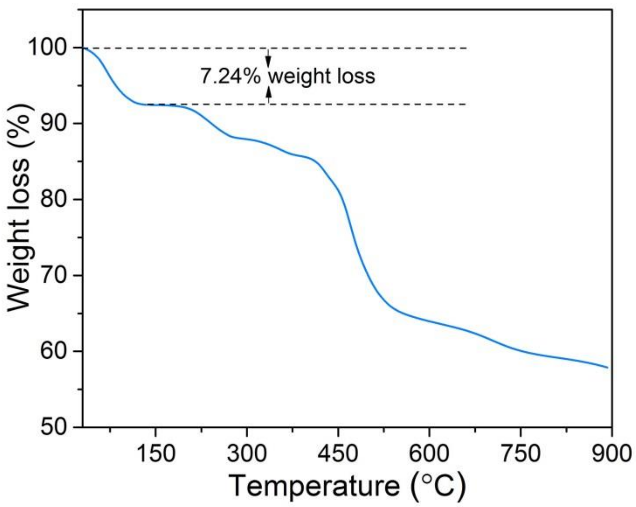

Supplementary Figure 11. The thermogravimetric analysis data showing that Th-SINAP-100 is thermally stable up to 150 °C.

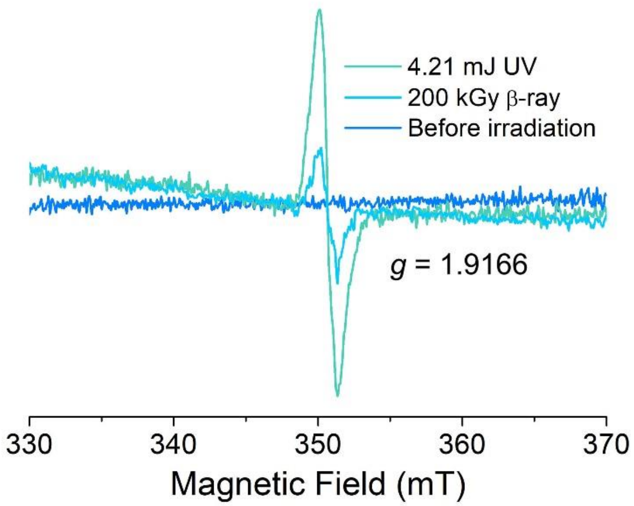

Supplementary Figure 12. EPR spectra of HTPC before and after UV and β-ray irradiation.

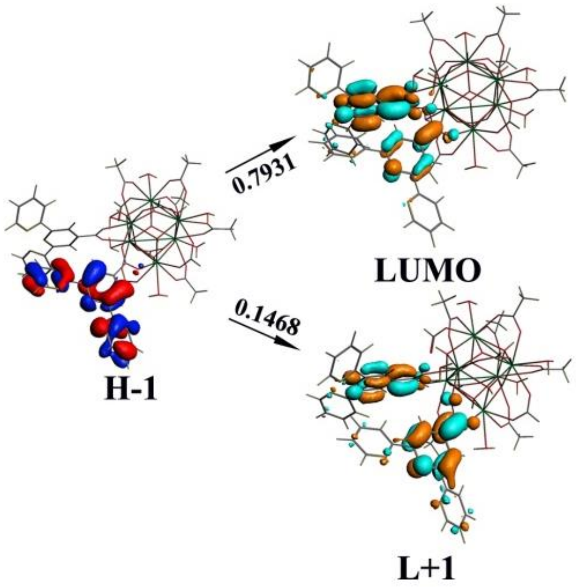

Supplementary Figure 13. Orbital diagrams of the 392 nm electron transitions for TS-d1 from the TD-DFT calculations.

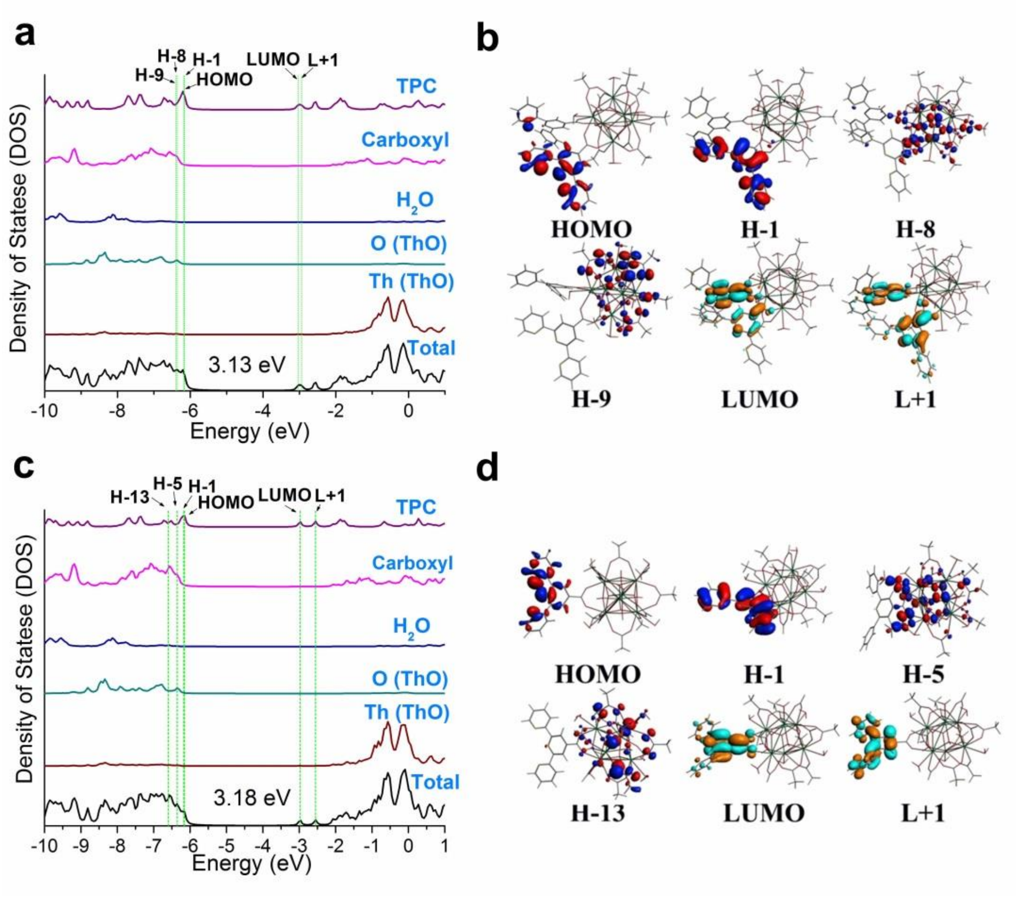

Supplementary Figure 14. (a) Density of States (DOS) of TS-d1. (b) The diagrams of selected orbitals of TS-d1. (c) Density of States (DOS) of TS-m1. (d) The diagrams of selected orbitals of TS-m1.

Supplementary Tables

Supplementary Table 1. Crystallographic data for Th-SINAP-100 before and after γ-ray irradiation.

Sample	nonirradiated	irradiated
Mass	4006.47	4006.47
Color	Purple	Yellow
Habit	Block	Block
Space group	$P\bar{1}$	$P\bar{1}$
a (Å)	14.5258(11)	14.500(6)
b (Å)	17.2459(16)	17.173(7)
c (Å)	18.5038(15)	18.368(9)
α (°)	107.681(3)	106.984(15)
β (°)	99.357(3)	99.480(16)
γ (°)	111.482(3)	111.525(16)
V (Å^3)	3908.7(6)	3875(3)
Z	1	1
T (K)	293(2)	293(2)
λ (Å)	0.71073	0.71073
Max 2θ (°)	55.188	55.174
ρ_{calcd} (g cm^{-3})	1.702	1.717
μ (Mo Ka)	0.71073	0.71073
R_1	0.0239	0.0330
wR_2	0.0665	0.0781
R_{int}	0.0429	0.0531
GOF	0.984	0.987

Supplementary Table 2. The CIE chromaticity coordinates (*x*, *y*) of PL upon irradiation with different UV dose for Th-SINAP-100 and HTPC.

Dosage (mJ)	Th-SINAP-100 CIE coordinates (*x*, *y*)	Dosage (mJ)	Ligand CIE coordinates (*x*, *y*)
0	(0.21,0.23)	0	(0.16,0.11)
1.17×10^{-3}	(0.21,0.25)	2.99×10^{-1}	(0.16,0.15)
2.91×10^{-3}	(0.21,0.26)	9.97×10^{-1}	(0.17,0.19)
5.85×10^{-3}	(0.21,0.28)	2.72	(0.18,0.25)
1.17×10^{-2}	(0.21,0.30)	4.79	(0.19,0.27)
1.75×10^{-2}	(0.21,0.32)	7.17	(0.19,0.29)
3.51×10^{-2}	(0.22,0.37)	11.24	(0.21,0.32)
1.40×10^{-1}	(0.23,0.40)	15.38	(0.21,0.33)
3.16×10^{-1}	(0.26,0.44)	25.82	(0.23,0.36)
5.26×10^{-1}	(0.28,0.46)	41.44	(0.25,0.38)
7.02×10^{-1}	(0.30,0.47)	47.50	(0.25,0.39)
8.77×10^{-1}	(0.30,0.48)		
1.05	(0.30,0.48)		
1.23	(0.31,0.49)		
1.44	(0.31,0.49)		
1.75	(0.31,0.50)		
2.11	(0.32,0.49)		
2.81	(0.32,0.49)		
3.51	(0.32,0.49)		
4.21	(0.31,0.49)		

Supplementary Table 3. The comparison of γ and β radiation resistance in MOFs.

MOFs	β irradiation	γ irradiation	Reference
Th-SINAP-100	1 MGy	1 MGy	This work
$[(CH_3)_2NH_2][UO_2(L1)]$	200 kGy	N/A	1
SCU-100	100 kGy	N/A	2
SCU-101	200 kGy	N/A	3
FJSM-InMOF	200 kGy	N/A	4
$\{[Ni_3Th_6(\mu_3\text{-}O)_4(\mu_3\text{-}OH)_4(IN)_{12})(H_2O)_{12}]\cdot(OH)_6\cdot 5DMF\cdot 2H_2O\}_n$	400 kGy	100 kGy	5
SCU-200	N/A	200 kGy	6
SIFSIX-3-Cu	50 kGy	50 kGy	7
MIL-100(Al)	N/A	2000 kGy	8

Supplementary Table 4. Selected bond distances (Å) of Th-SINAP-100 before and after γ-ray irradiation**.**

Assignment	Bond	nonirradiated	irradiated
Th−O_W	Th1−O1	2.643(3)	2.647(4)
	Th2−O5	2.703(4)	2.711(6)
	Th3−O13	2.686(3)	2.679(4)
Th−$O_{oxo/hydroxo}$	Th1−O3	2.378(3)	2.382(4)
	Th2−O3	2.387(3)	2.390(4)
	Th3−O3	2.373(3)	2.381(4)
	Th1−O9A/Th1−O9B	2.351(5)/2.468(6)	2.357(8)/2.474(11)
	Th2−O9A/Th2−O9B	2.217(6)/2.531(7)	2.242(9)/2.515(12)
	Th3−O9A/Th3−O9B	2.288(5)/2.505(6)	2.284(9)/2.523(11)
	Th1−O16A/Th1−O16B	2.341(5)/2.528(6)	2.335(7)/2.573(9)
	Th2−O16A/Th2−O16B	2.302(5)/2.510(6)	2.276(7)/2.544(9)
	Th3−O16A/Th3−O16B	2.252(5)/2.567(6)	2.293(7)/2.542(10)
	Th1−O18A/Th1−O18B	2.250(6)/2.590(8)	2.401(5)
	Th2−O18A/Th2−O18B	2.350(6)/2.448(6)	2.374(4)
	Th3−O18A/Th3−O18B	2.317(6)/2.488(6)	2.379(4)
Th−O_{TPC}	Th1−O2	2.559(3)	2.564(4)
	Th2−O4	2.432(3)	2.430(4)
	Th2−O6	2.468(3)	2.460(4)
	Th1−O7	2.507(3)	2.510(4)
	Th2−O8	2.518(3)	2.520(4)
	Th3−O10	2.474(3)	2.477(4)
	Th1−O11	2.492(3)	2.496(4)
	Th3−O12	2.477(3)	2.475(4)
Th−O_{HCOO}	Th3−O14	2.505(3)	2.508(4)
	Th2−O15	2.515(3)	2.505(4)
	Th1−O17	2.509(3)	2.515(4)
	Th3−O19	2.554(3)	2.565(4)
π-π stacking distance	TPC−TPC (N10,11,12)	3.178	3.160

Supplementary Table 5. Optimized bond lengths (Å) for the experimentally-synthesized complex (TS) and three theoretical models (TS-m1 and TS-d1), compared with experimental values.

		Th-$O_{oxo/hydroxo}$ [a]	Th-O_{TPC}	Th-O_{HCOO}	Th-O_{W}
TS	Calc.	2.278-2.539	2.469-2.568	2.420-2.600	2.720-2.754
		(2.413) [b]	(2.493)	(2.515)	(2.735)
	Expt.	2.332-2.399	2.458-2.528	2.422-2.554	2.644-2.691
		(2.383)	(2.497)	(2.466)	(2.672)
TS-m1	Calc.	2.293-2.537	2.461-2.590	2.442-2.577	2.717-2.735
		(2.421)	(2.526)	(2.490)	(2.730)
TS-d1	Calc.	2.288-2.735	2.449-2.573	2.306-2.593	2.517-2.747
		(2.445)	(2.490)	(2.485)	(2.660)

[a] Four types of oxo atoms include $O_{oxo/hydroxo}$ (thorium cluster), O_{TPC} (TPC ligand), O_{HCOO} (HCOO ligand), and O_{W} (water).

[b] The average value in parentheses

Supplementary Table 6. Bond orders of the experimentally-synthesized complex (TS) and two theoretical modes (TS-m1 and TS-d1).

	Th-$O_{oxo/hydroxo}$	Th-O_{TPC}	Th-O_{HCOO}	Th-O_{W}
TS	0.40-0.80	0.45-0.56	0.34-0.39	0.42-0.57
	(0.59) [a]	(0.53)	(0.37)	(0.49)
TS-m1	0.41-0.77	0.44-0.53	0.39	0.45-0.58
	(0.58)	(0.49)	(0.39)	(0.53)
TS-d1	0.41-0.76	0.45-0.55	0.38-0.39	0.43-0.58
	(0.59)	(0.52)	(0.39)	(0.50)

[a] The average value in parentheses

Supplementary Table 7. Calculated absorptions of the molecular models and TPC ligand at the TD-DFT level.

	λ(nm)[a]	λ(nm)[b]	E(eV)[b]	*f* [c]	Configuration	Weight > 0.1	Expt.
TS-d1	392	392	3.16	0.0085	H-1→LUMO	0.7931	410
					H-1→L+1	0.1468	
	360	366	3.39	0.0380	H-5→L+1	0.5211	340
					H-6→L+1	0.2232	
		364	3.41	0.1504	H-1→L+1	0.4127	
					H-7→L+1	0.2443	
		363	3.42	0.0528	H-7→L+1	0.7383	
					H-1→L+1	0.1345	
		361	3.43	0.0532	H-10→LUMO	0.3368	
					H-8→L+1	0.2505	
					H-4→L+1	0.1118	
		360	3.45	0.0251	H-10→LUMO	0.6183	
					H-8→L+1	0.1303	
		357	3.47	0.1019	H-8→L+1	0.5451	
TS-m1	382	382	3.25	0.0035	HOMO→LUMO	0.9119	410
	365	367	3.38	0.0484	H-5→LUMO	0.7312	340
					H-1→LUMO	0.2345	
		365	3.40	0.1971	H-1→LUMO	0.6760	
					H-5→LUMO	0.2560	
TPC	400	401	3.09	0.0138	H-1→LUMO	0.9820	406
	394	394	3.15	0.0201	H-1→L+1	0.9911	373
	343	343	3.62	0.2397	H-2→LUMO	0.9034	342
		332	3.74	0.0459	H-4→LUMO	0.4783	
					H-2→L+1	0.2027	
					H-5→L+1	0.1273	
	282	323	3.83	0.1092	H-4→L+1	0.5832	291
					H-5→LUMO	0.3094	
		312	3.98	0.1139	H-5→L+1	0.4961	
					H-2→L+1	0.3366	
		283	4.38	0.1693	H-7→LUMO	0.7412	
		282	4.39	0.5424	H-7→L+1	0.6908	
					H-2→L+3	0.1121	
		264	4.70	0.1561	H-2→L+4	0.6100	
					H-3→L+4	0.1823	

262	4.73	0.0689	H-3→L+4	0.7634
			H-2→L+4	0.1146
258	4.81	0.0385	H-9→LUMO	0.4605
			H-8→L+1	0.2398
			H-6→L+3	0.2127
250	4.96	0.0771	H-7→L+2	0.4723
			HOMO→L+5	0.2058
			H-10→L+1	0.1124

[a] The simulated absorption band (nm).
[b] Calculated absorption transitions in nm, eV and cm^{-1}.
[c] Oscillator strength (*f*), which was multiplied by 100.

Supplementary References

1. Wang, Y., *et al.* Umbellate Distortions of the Uranyl Coordination Environment Result in a Stable and Porous Polycatenated Framework That Can Effectively Remove Cesium from Aqueous Solutions. *J. Am. Chem. Soc.* **137**, 6144–6147 (2015).

2. Sheng, D., *et al.* Efficient and Selective Uptake of TcO_4^- by a Cationic Metal–Organic Framework Material with Open Ag^+ Sites. *Environ. Sci. Technol.* **51**, 3471–3479 (2017).

3. Zhu, L., *et al.* Identifying the Recognition Site for Selective Trapping of $^{99}TcO_4^-$ in a Hydrolytically Stable and Radiation Resistant Cationic Metal–Organic Framework. *J. Am. Chem. Soc.* **139**, 14873-14876 (2017).

4. Gao, Y.-J., Feng M.-L., Zhang B., Wu Z.-F., Song Y., Huang X.-Y. An easily synthesized microporous framework material for the selective capture of radioactive Cs^+ and Sr^{2+} ions. *J. Mater. Chem. A* **6**, 3967-3976 (2018).

5. Xu, H., *et al.* High Uptake of ReO_4^- and CO_2 Conversion by a Radiation-Resistant Thorium–Nickle [$Th_{48}Ni_6$] Nanocage-Based Metal–Organic Framework. *Angew. Chem. Int. Ed.* **58**, 6022-6027 (2019).

6. Liu, H., *et al.* Emergence of Radical-Stabilizing Metal-Organic Framework as a New Type of Radio-Photoluminescence Dosimeter. *Angew. Chem. Int. Ed.* **59**, 1-7 (2020).

7. Elsaidi, S.K., *et al.* Radiation-resistant metal-organic framework enables efficient separation of krypton fission gas from spent nuclear fuel. *Nat. Commun.* **11**, 3103 (2020).
